# Methods for Communicating the Impact of Parameter Uncertainty in a Multiple-Strategies Cost-Effectiveness Comparison

**DOI:** 10.1177/0272989X221100112

**Published:** 2022-05-19

**Authors:** Henri B. Wolff, Venetia Qendri, Natalia Kunst, Fernando Alarid-Escudero, Veerle M.H. Coupé

**Affiliations:** Department of Epidemiology and Data Science, Amsterdam UMC, Vrije Universiteit Amsterdam, Amsterdam Public Health, Amsterdam, the Netherlands; Department of Epidemiology and Data Science, Amsterdam UMC, Vrije Universiteit Amsterdam, Amsterdam Public Health, Amsterdam, the Netherlands; Department of Epidemiology and Data Science, Amsterdam UMC, Vrije Universiteit Amsterdam, Amsterdam Public Health, Amsterdam, the Netherlands; Department of Health Management and Health Economics, Faculty of Medicine, University of Oslo, Oslo, Norway; Cancer Outcomes, Public Policy and Effectiveness Research (COPPER) Center, Yale University School of Medicine and Yale Cancer Center, New Haven, CT, USA; Public Health Modeling Unit, Yale University School of Public Health, New Haven, CT, USA; Division of Public Administration, Center for Research and Teaching in Economics (CIDE), Aguascalientes, AGS, Mexico, MX-AGU, Mexico; Department of Epidemiology and Data Science, Amsterdam UMC, Vrije Universiteit Amsterdam, Amsterdam Public Health, Amsterdam, the Netherlands

**Keywords:** CEA, CEAC, ELC, many strategies, PSA, sensitivity analysis

## Abstract

**Purpose:**

Analyzing and communicating uncertainty is essential in medical decision making. To judge whether risks are acceptable, policy makers require information on the expected outcomes but also on the uncertainty and potential losses related to the chosen strategy. We aimed to compare methods used to represent the impact of uncertainty in decision problems involving many strategies, enhance existing methods, and provide an open-source and easy-to-use tool.

**Methods:**

We conducted a systematic literature search to identify methods used to represent the impact of uncertainty in cost-effectiveness analyses comparing multiple strategies. We applied the identified methods to probabilistic sensitivity analysis outputs of 3 published decision-analytic models comparing multiple strategies. Subsequently, we compared the following characteristics: type of information conveyed, use of a fixed or flexible willingness-to-pay threshold, output interpretability, and the graphical discriminatory ability. We further proposed adjustments and integration of methods to overcome identified limitations of existing methods.

**Results:**

The literature search resulted in the selection of 9 methods. The 3 methods with the most favorable characteristics to compare many strategies were 1) the cost-effectiveness acceptability curve (CEAC) and cost-effectiveness acceptability frontier (CEAF), 2) the expected loss curve (ELC), and 3) the incremental benefit curve (IBC). The information required to assess confidence in a decision often includes the average loss and the probability of cost-effectiveness associated with each strategy. Therefore, we proposed the integration of information presented in an ELC and CEAC into a single heat map.

**Conclusions:**

This article presents an overview of methods presenting uncertainty in multiple-strategy cost-effectiveness analyses, with their strengths and shortcomings. We proposed a heat map as an alternative method that integrates all relevant information required for health policy and medical decision making.

**Highlights:**

- To assess confidence in a chosen course of action, decision makers require information on both the probability and the consequences of making a wrong decision.
- This article contains an overview of methods for presenting uncertainty in multiple-strategy cost-effectiveness analyses.
- We propose a heat map that combines the probability of cost-effectiveness from the cost-effectiveness acceptability curve (CEAC) with the consequences of a wrong decision from the expected loss curve.
- Collapsing of the CEAC can be reduced by relaxing the CEAC, as proposed in this article.
- Code in Microsoft Excel and R is provided to easily analyze data using the methods discussed in this article.

## Introduction

Cost-effectiveness analysis (CEA) is a method that compares the costs and health benefits of alternative strategies, allowing policy makers to make informed decisions. The optimal strategy often depends on the willingness to pay (WTP) per unit of health gain. The confidence in the chosen course of action should be assessed in sensitivity analyses to determine how parameter uncertainty can affect model outcomes. Validation studies can also be used to determine how good the conclusions hold for different patient populations and tools to identify other methodological issues such as structural uncertainty.^1,2^

Probabilistic sensitivity analysis (PSA) is a powerful method to assess parameter uncertainty and is therefore an essential requirement for health technology assessments in many journals, guidelines, and reimbursement or funding agencies.^3,4^ PSAs are performed to propagate uncertainty from model input parameters to model outcomes and give insight into the impact of uncertainty around model parameters on health and cost outcomes of different decision options. A PSA is conducted by randomly sampling model parameters’ values from prespecified distributions and reestimating the model outcomes.

Traditionally, when 2 strategies are compared, the PSA can be presented in a cost-effectiveness plane by plotting the difference in costs and the difference in effectiveness between the 2 strategies. This gives insight into 2 important features: the percentage of simulations in which the new strategy is cost-effective compared with the comparator strategy and the size of the differences in costs and effectiveness. A strategy can have a high probability of being cost-effective, but this probability may be less important if the differences in costs and effectiveness are not relevant in size. Both the probability of cost-effectiveness and the comparator strategy that can potentially be lost when a wrong decision is made should therefore be included in a CEA and the decision-making process of policy makers.

It is, however, unclear how to interpret a cost-effectiveness plane with more than 2 strategies.^5^ PSA outcomes can also be presented graphically with other methods, differing in the type of information shown, which is often a probability, risk, benefit, or loss assigned to a wrong decision. CEA guidelines advise presenting the PSA results using the cost-effectiveness acceptability curve (CEAC) and frontier (CEAF). The CEAC shows the probability of cost-effectiveness for each strategy and the CEAF the strategy with the highest expected net benefit, but neither shows the consequences of making a wrong decision.^1,6,7^

Furthermore, if a study involves a comparison of a high number of strategies, it is likely that many of the strategies’ outcomes are relatively similar. In this case, the probability for any single strategy to outperform all other comparator strategies may be low, leading to overlapping CEACs with a very low probability of any strategy being cost-effective. An example of this problem is visible in the study by Wolff et al.,^8^ which compared 108 surveillance strategies of lung cancer, resulting in interpretation problems for decision makers.^9^ In these types of surveillance and screening studies that compare many strategies with very similar outcomes, other factors may become important in the choice for the “best” strategy, such as the difficulty in implementing a strategy. To our knowledge, there is no consensus on the best way to represent and communicate the impact of parameter uncertainty in economic evaluations when considering many alternative strategies.

Hence, in the present study, we first performed a citation-mining literature search to identify alternative methodologies to the CEAC and CEAF to represent uncertainty.^10^ Second, we identified potential strengths and shortcomings for each of the identified methods by applying them to 3 PSA data sets from different, previously published decision-analytic models that considered many strategies. Third, we propose an approach to address some of the potential shortcomings of the existing methods by modifying and integrating the identified methods. Finally, we provide the R code developed to apply the identified methods.

## Methods

### Systematic Review

The current standard methods for graphical representation of uncertainty recommended by the Professional Society for Health Economics and Outcomes Research–Society for Medical Decision Making (ISPOR-SMDM) Task Force are the CEAC and the CEAF.^1^

We performed a systematic search of the literature to identify other methods that can assess and communicate the impact of uncertainty in a cost-effectiveness comparison of multiple strategies. We used a forward snowballing approach^10^ between March 4, 2020, and August 26, 2020, to identify articles that cited articles in which the CEAC “OR” CEAF were introduced,^11,12^ using the “see all cited by articles” function on PubMed.

**Figure 1.**
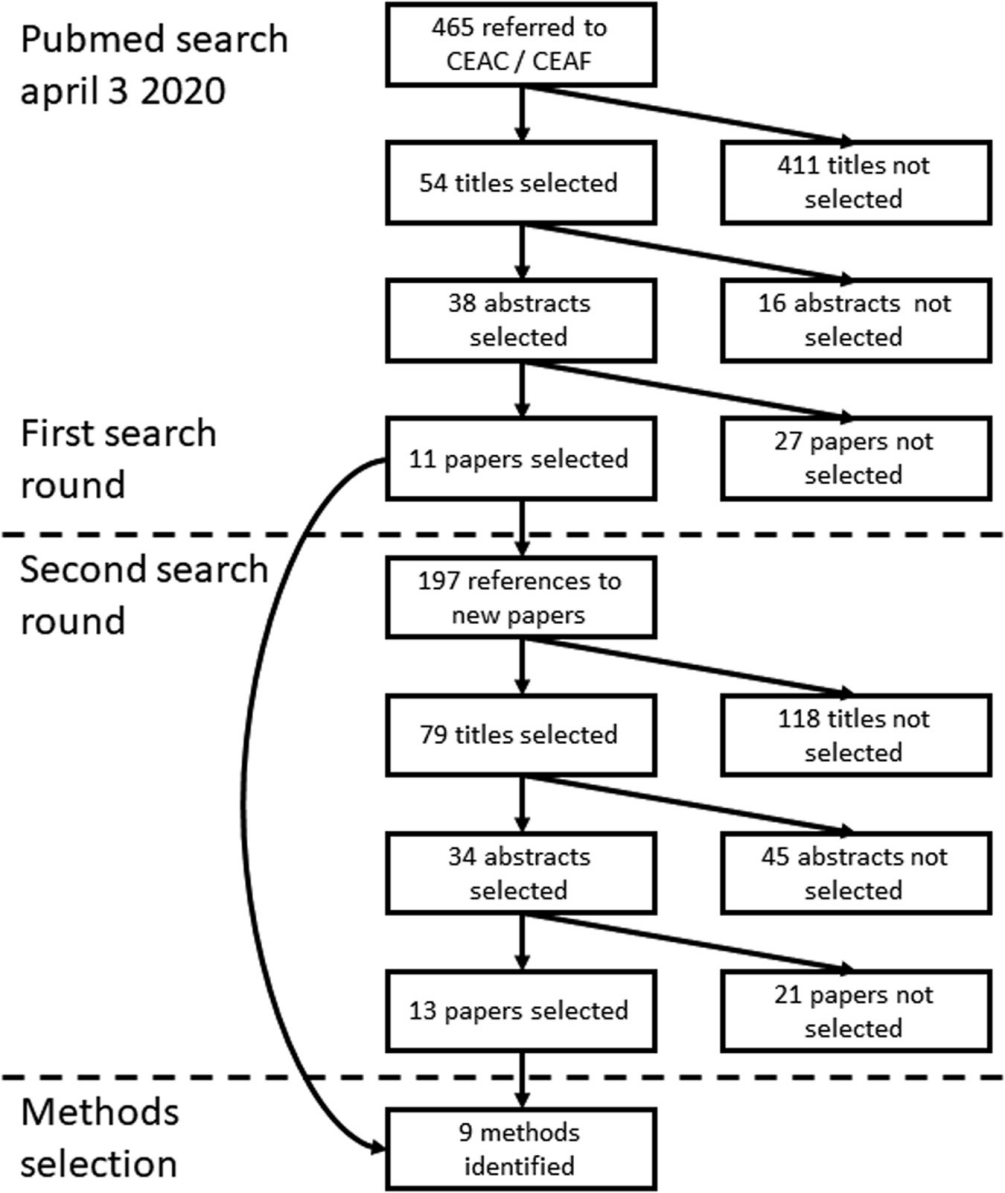
Flow chart of the systematic literature search. The first search round used forward snowballing to identify the titles of articles that referenced the original articles introducing the methodology of the cost-effectiveness acceptability curve (CEAC) and cost-effectiveness acceptability frontier (CEAF). The second search round used backward snowballing to identify articles referenced by articles selected in the first search round. In both the first and second search rounds, the selection criteria were applied to titles, abstracts, and full papers. Nine methods were identified from the 24 selected articles.

The identified articles were reviewed in the first search round based on titles and subsequently on abstracts and full texts by 2 reviewers (H.B.W. and V.Q. or V.M.H.C.). If the reviewers disagreed on inclusion/exclusion, a third reviewer was consulted (V.Q., V.M.H.C., or N.K.). In the second search round, we used backward snowballing^10^ to review all citations in the articles that were selected in the first round (Figure 1).

The selection criteria for titles, abstracts, and full text were as follows:

1. Articles that include methods representing the probability or potential consequences of all potential outcomes associated with the selection of the cost-effective strategy from multiple options were included.
2. Methods that cannot be applied to a PSA data set were excluded, as they cannot be compared with other methods by application to case studies.
3. If multiple articles discussed the same method (for instance, the CEAC), only the article with the earliest publication date was selected.
4. Only articles written in English were selected for review.

Value-of-information analysis (VOI) methods were excluded, because VOI does not compare the probability of cost-effectiveness or monetary or health losses related to the potential consequences of multiple options and instead focuses on the uncertainty of the efficient frontier. Therefore, VOI lies outside of the scope of the current study. For more information on this topic, we refer to other review articles on VOI.^7,13,14^

### Application of Methods

The identified methods that are used to present the uncertainty in CEAs are evaluated with the use of PSA data sets. A PSA is designed to reflect the effect of the underlying parameter uncertainty on the conclusions of a model. In a PSA, model simulations are run iteratively using different parameter sets that have been randomly drawn from their respective distributions. For this methods comparison, PSA data sets of 3 case studies were used.

The first example PSA data set came from the study of Rojnik et al.^15^ Methods were also applied to 2 additional case studies with 5 and 108 strategies to investigate the effect of the number of compared strategies on graphical discriminatory ability (see Appendix 2). The authors from the 3 studies provided a file with the PSA output from their respective analysis, which contained the costs and life-years or quality-adjusted life-years (QALYs) of each strategy considered in their analysis and for each PSA iteration.

The study by Rojnik et al.^15^ compared the costs and health effects of 37 breast cancer prevention strategies for a healthy population, based on mammography screening with 3 intervals over 12 different screening periods and 1 strategy with no screening. Breast cancer can be diagnosed as local, regional, or distant in the model, resulting in different probabilities of breast cancer death. Breast cancer can also be clinically detected after symptoms appear. A Markov cohort model was used, for which 38 input model parameters were varied in the PSA. See Appendix section 2 for a description and implementation of the other 2 case studies.

In the systematic review, we focused on methods that can be applied to different cost-effectiveness measures that integrate the costs and health outcomes of each considered strategy, including the net monetary benefit (NMB), net health benefits, and return on investment. These are calculated as NMB = WTP × Effectiveness – Costs, NHB = Effectiveness – Costs/WTP, and ROI = (WTP × Effectiveness – Costs)/Costs. For the case studies, we used the NMB because it is the most commonly used measure. To translate the health effect (QALYs or life-years) into monetary value, we used either a variable WTP or a fixed WTP of 50,000 €/QALY.^16^

### Comparison and Adaptation of Methods

We compared all methods identified through the literature review focusing on the following 4 characteristics:

1. The type of uncertainty information conveyed. Uncertainty can either be expressed as a probability related to a specified outcome or an outcome value such as NMB, incremental NMB, or the variable “expected loss,” which is the average difference in NMB with the cost-effective strategy.
2. Interpretability of the graphical representation, scored by the authors of this study. We gave a score between 1 and 10 based on the response to 3 questions: “I understand the variables plotted on the *x*-axis and *y*-axis of the figure,” “This figure will help me in making a decision,” and “This figure clearly shows the probability or consequences related to the decision options.” These scores were subsequently averaged over questions and authors. Average scores below 6 are considered “bad,” between 6 and 8 are “average,” and 8 and above are “good.”
3. WTP threshold. Whether the impact of uncertainty is assessed over a range of WTP threshold values (represented on the *x*-axis) or fixed at a specific WTP threshold.
4. Graphical discriminatory ability. We assigned a score of “good” when all lines are visible, “bad” when less than half of the lines are visible, or when the cost-effective strategies are indistinguishable by eye, and “average” when discriminatory ability lies in between those two.

In comparing the methods, we used the identified shortcomings to make suggestions for improvement of the current methods. These adjustments are discussed in the Results section “adapted methods” and further discussed in Appendix section 3.

## Results

### Systematic Review

The first round of the literature search resulted in 465 records on PubMed. Based on our selection criteria, 54 titles were selected. Excluding all abstracts with no full articles identified led to the exclusion of 16 titles. For the remaining 38 titles, the full-text articles were evaluated, which resulted in the selection of 11 articles. The references of these 11 articles provided 197 unique new articles that were reviewed in the second round. A total of 79 articles were selected from those articles, of which 34 abstracts were evaluated, resulting in 13 full-text articles. The combination of the 11 and 13 full-text articles that were selected jointly described 9 unique methods (Figure 1).

### Application to Case Studies and Characteristics of the Methods

The 9 methods and their characteristics are shown in Table 1 and discussed below. The methods are briefly described in the following paragraphs, and the formulas used to construct each of the graphical representations can be found in Appendix section 1.

**Table 1:** Comparison of Identified Methods^a^

Method Name	Type of Information	Interpretability	Variable WTP	Graphical Discriminatory Ability with Number of Strategies			References
				3	37	108	
Cost-effectiveness acceptability curve + frontier (CEAC/CEAF)	Probability of cost effectiveness	+	Yes	+	+/−	−	^11,12^
Expected loss curve (ELC)	Expected loss	+	Yes	+	+	+	^17^
Expected benefit plot	NMB + 95% interval	−	Yes	−	−	−	^16^
Net benefit density plot	Probability of NMB + NMB	−	No	+	−	−	^18^
Incremental benefit density plot	Probability of incremental NMB + incremental NMB	−	No	+	+/−	−	^18^
Stochastic dominance	Cumulative probability of NMB + NMB	+/−	No	+	+/−	−	^16^
Incremental benefit curve	Cumulative probability of incremental NMB + incremental NMB	+/−	No	+	+/−	+/−	^19^
Return-risk space	Mean NMB + standard deviation NMB	−	No	+	+	+	^20^
Cumulative rankogram	Cumulative probability of rank NMB + rank NMB	−	No	−	−	−	^21^

a Characteristics of the graphical methods for representing the impact of decision uncertainty. The type of uncertainty information can either be a probability or a variable used to illustrate differences in net monetary benefit (NMB). In the third column, interpretability is scored as good, average, or bad based on a short questionnaire filled out by each author (see the “Methods” section). The fourth column states whether a range of willingness-to-pay (WTP) threshold values are considered (on the *x*-axis of the plot; Yes) or whether a fixed WTP threshold value (No) is used. Graphical discriminatory ability as observed from the application of these methods to the 3 probabilistic sensitivity analysis data sets comparing 3, 37, and 108 strategies was scored as “good” (+) if all lines in the graph are clearly visible, “bad” (–) if less than half of the lines are clearly visible or if no distinction is possible among the most cost-effective strategies, and average otherwise (+/–; see the “Results” section and Appendix section 2 for application of the methods to the data sets). The final column contains references to articles where the methods are described.

We refer to Github^22^ for the R code that can be used to make the graphs corresponding to each of the methods for each case study and for the Excel file that can be used to create a CEAC, expected loss curve (ELC), stochastic dominance plot, incremental benefit curve, and return risk plot.

### Methods with a WTP Axis

**Figure 2:**
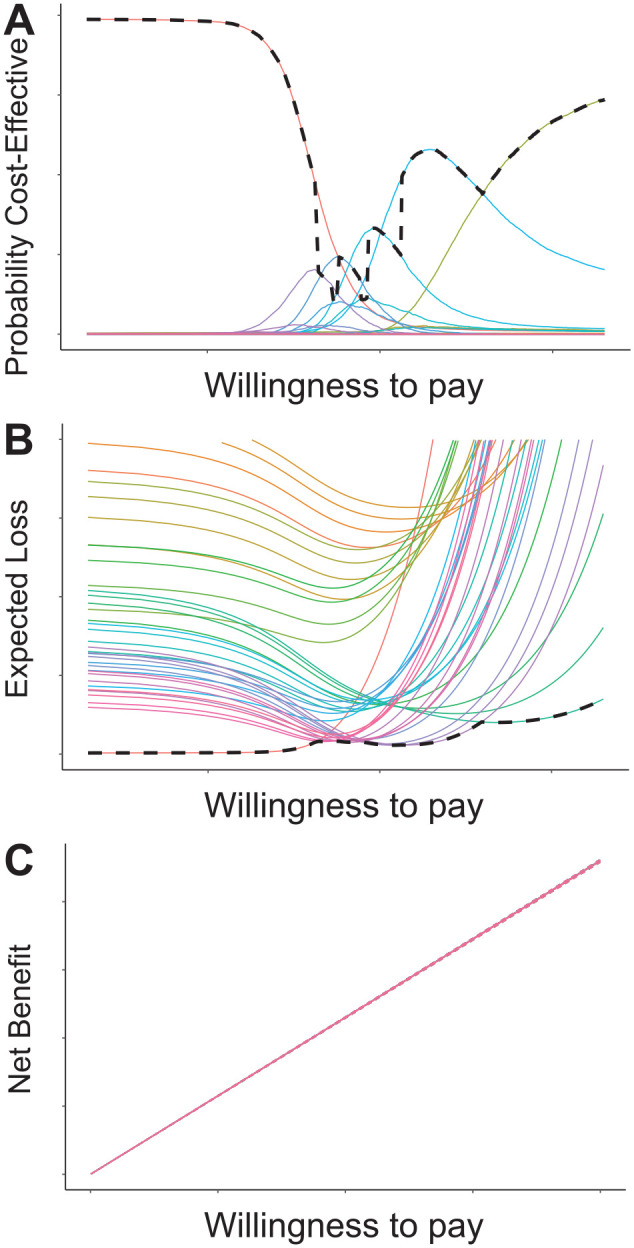
Illustrative comparison of methods to communicate the impact of uncertainty with a willingness-to-pay axis. (A) The cost-effectiveness acceptability curve (CEAC) and its frontier (dashed black line).^11,12^ (B) The expected loss curves (ELCs) and their frontiers (dashed black line).^17^ (C) The expected benefit plot with upper and lower limit of the 95% prediction interval (dashed lines).^16^ All 3 methods use the *x*-axis to depict a range of willingness-to-pay threshold values, whereas the *y*-axis is used to show probabilities of cost-effectiveness for the CEAC, expected loss values for the ELC, and net monetary benefit for the expected benefit plot. The frontiers show which strategies have the highest expected net monetary benefit.

Figure 2A–C shows the application of the CEAF, ELC, and expected benefit plot, respectively. In all 3 methods, the *x*-axis shows the WTP threshold used to calculate the NMB values for each strategy in the PSA. The dashed black lines show the frontiers in the CEAC and ELC, which are the strategies with the highest expected NMB.

The CEAC shows the probability of each strategy being cost-effective on the y-axis, which is the proportion of PSA iterations that each strategy has the highest NMB value. The CEAC shows in which WTP regions there is less certainty that the strategies with the highest NMB are cost-effective. For instance, in figure 2A the third strategy on the frontier has a probability of cost-effectiveness less than 20%. However, given that the CEAC counts only the times that a strategy has the highest NMB in a PSA iteration, strategies that have only minimally lower NMB are not identified.

The ELC shows the expected loss values on the *y*-axis, which is the difference between the NMB of a strategy and the maximum NMB reached in each iteration of the PSA. The ELC depicts the expected loss values over all PSA rounds. For instance, in Figure 2B, the second best option has a difference in expected loss of at most €250 NMB.

The expected benefit plot depicts the expected NMB of all strategies as a function of WTP, and the 2.5th and 97.5th percentiles of the NMB distribution (dashed lines).

A shortcoming of the CEAC is that it collapses when many strategies are compared. Only the better-performing strategies can be graphically distinguished in Figure 2A. Unlike the CEAC, the ELC does not collapse and is robust to the number of strategies compared (see Appendix 2). However, the expected benefit plot is not usable because the lines corresponding to the different strategies and their upper and lower bounds are highly condensed and have become indistinguishable. This may be caused by the extremely large differences between NMB values corresponding to the minimum and maximum WTP, compared with relatively small differences between strategies. This limits the option to zoom in on small differences between strategies. Changing the axis in the expected benefit plot to a logarithmic axis does not increase the graphical discriminatory ability (result not shown). The expected benefit plot contains information similar to the ELC, but the ELC is better at graphically distinguishing the curves because losses are less affected by WTP. As a result, the differences between strategies cannot be distinguished in Figure 2C.

### Methods with a Fixed WTP Threshold

**Figure 3:**
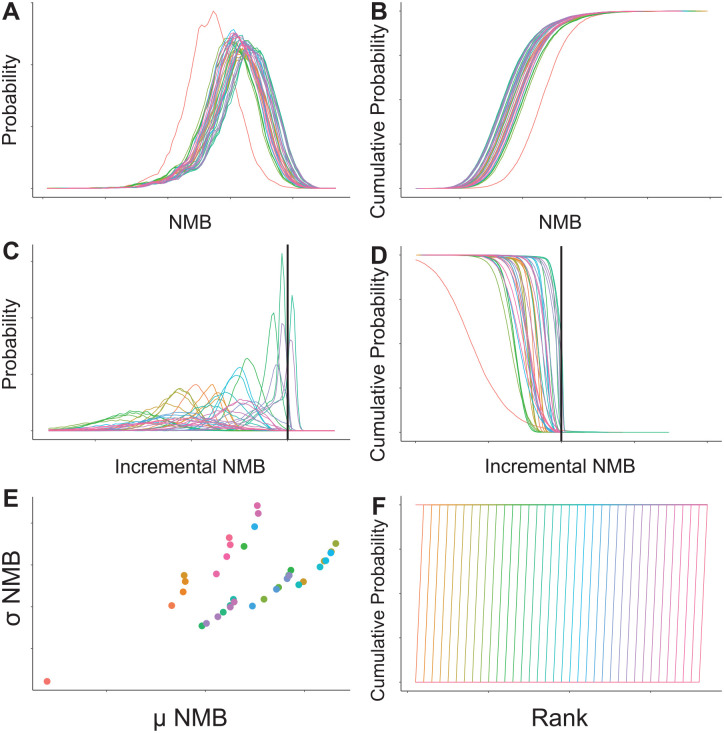
Illustrative comparison of methods that communicate the impact of uncertainty with a fixed willingness-to-pay threshold. The methods are (A) net benefit density plot,^18^ (B) stochastic dominance plot,^16^ (C) incremental benefit density plot,^18^ (D) incremental benefit curve,^19^ (E) return-risk space,^20^ and (F) cumulative rankogram.^21^ To produce these plots, the willingness-to-pay threshold was fixed at 50,000 €/quality-adjusted life-year for all figures. The probability density plots are normalized smoothened histograms of the net monetary benefits, using 100 and 500 bins for A and C, and the smoothening parameter was set at 0.5 (see Appendix 1C,D for the smoothening algorithm).

Figure 3 depicts the 6 graphical methods that give insight into the distributions of NMB or incremental NMB: the net benefit density plot, stochastic dominance, incremental benefit density plot, incremental benefit curve, return-risk space, and cumulative rankogram. The net benefit density, stochastic dominance plots, and return-risk space are based on NMB, whereas the incremental benefit density plot and incremental benefit curve consider incremental NMB, and the cumulative rankogram uses ranked NMB. A fixed WTP threshold was used to calculate all NMB values (50,000 €/QALY for Figure 3). This threshold is arbitrary and does not affect the interpretability or graphical discriminatory ability.

The methods in Figure 3 all show NMB or a variable related to NMB on the *x*-axis and the probability linked to that NMB variable on the *y*-axis. The net benefit density plot and incremental benefit density plot in Figure 3A and C are probability density plots; thus, the *y*-axis shows the relative likelihood of having NMB corresponding to the NMB values on the *x*-axis. The strategy with the highest area under the curve within a specific NMB range has the highest probability of having a NMB within that range. A shortcoming of the probability density plots is that it approximates the probability distribution from a PSA sampling of the underlying distribution by normalizing a smoothened histogram. This normalization requires choices such as the width of the bars and the smoothening method (Appendix 1C,D), which affect the shape of the curves and may introduce potential bias, affecting both the interpretation and interpretability when the curves lie close to each other. However, plotting cumulative density functions does not require similar choices, facilitating their interpretability.

Figures 3B and D are the cumulative probability versions of Figures 3A and C. Stochastic dominance plots, incremental benefit curves, and cumulative rankograms (Figure 3B,D,F) are cumulative density plots and show the probabilities of achieving NMB versus INB values greater or equal and smaller or equal than the value on the *x*-axis, respectively. This facilitates interpretability, making the plots easier to use. The strategy with the highest area under the curve has the highest expected NMB, in the case of the stochastic dominance plot, and the incremental benefit curve. Alternatively, policy makers can choose for a tradeoff between the strategies with the higher (incremental) NMB value and with lower probability of (incremental) NMB greater or equal to that specific value on the *x*-axis or strategies with the higher probability to reach a specific (incremental) NMB value.

Incremental NMB (used in Figure 3C,D) can be calculated for each PSA iteration, as the difference between the NMB of a strategy minus the maximum NMB of that iteration, unless the strategy has the highest NMB. In that case, the incremental NMB is the maximum NMB minus the second highest NMB. The vertical line in the incremental benefit density plot, and the incremental benefit curve is the point where the maximum and second highest NMB values are the same, thus showing the point where this rule changes. Therefore, incremental benefit highlights which strategies are cost-effective and how much higher their benefit is than the other strategies illustrated by the distance from the black line on the *x*-axis. For this reason, incremental benefit density plots and incremental benefit curves score slightly better than the net benefit density and stochastic dominance plots on interpretability and graphical discriminatory ability.

The return-risk space assumes that NMB is distributed normally, which is not always the case and is subject to verification. The mean and standard deviation of NMB of the strategies are plotted on the *x*-axis and *y*-axis, respectively. In the return-risk space, the standard deviation of NMB is interpreted as the uncertainty surrounding the NMB. However, compared with the net benefit density plot, the latter contains much more information on the distribution than the return-risk space and gives an idea as to whether the NMB distribution is normal or skewed.

**Figure 4:**
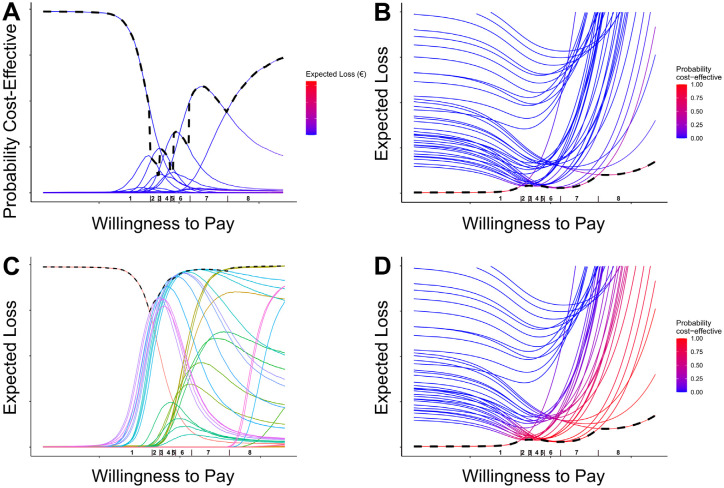
Adaptations of the cost-effectiveness acceptability curve (CEAC) and expected loss curve (ELC): the heat map and relaxed CEAC. The heat map in (A) shows the CEAC with expected loss values on the color scale (red representing high loss and blue low loss), and the heat map in (B) shows the ELC with the probability of being cost-effective on the color scale (red representing high probabilities and blue low probabilities). In (C), the graphical discriminatory ability of the CEAC is improved by relaxation of the CEAC. In the heat map in (D), graphical discriminatory ability of the color scale is improved by using the probability of being cost-effective of the relaxed CEAC on the ELC. The short vertical lines on the *x*-axis correspond to the incremental cost-effectiveness ratios of the strategies on the cost-effectiveness acceptability frontier, and the numbers denote which strategies are cost-effective in each interval of willingness-to-pay values. Strategies with a net monetary benefit (NMB) ≥99.95% of the maximum NMB value were considered cost-effective in the relaxed CEAC used for Figure 4C,D.

The cumulative rankogram is similar to the stochastic dominance plot but uses numerically ranked NMB scores for each strategy per PSA iteration. Rank numbers are shown on the *x*-axis from high to low (instead of NMB values). For the cumulative rankogram, 1 is the best-performing rank; thus, the cumulative probability corresponds to the probability that a strategy achieves a rank value smaller or equal to the value on the *x*-axis. The cumulative rankogram is extremely sensitive to the correlation in the underlying data, which results in the uninformative plot seen in Figure 4F. As the cumulative rankogram replaces NMB values with ranks, it does not provide the absolute NMB differences between strategies. Therefore, it provides less information about the uncertainty in the PSA than the stochastic dominance plot.

### Adapted Methods: The Relaxed CEAC and the Heat Map

The CEAC plots the probability of cost-effectiveness and its frontier (CEAF) shows which curves in the CEAC have the highest expected benefit values. The CEAC, however, does not provide information on the loss incurred when a wrong decision is taken. In contrast, the ELC provides information on the expected loss in NMB when a strategy is chosen that is not on its frontier. In addition, the frontier of the ECL corresponds to the expected value of perfect information.^1,9^ The ECL, on the other hand, does not inform about the probability of cost-effectiveness.

Both the CEAC and ELC provide valuable information required for well-balanced decision making. Thus, to inform policy makers, a graphical representation of the impact of uncertainty should preferably address these different perspectives. Therefore, we propose merging the information provided by both methods into a single heat map by integrating the CEAC and the ELC into 1 figure, using a color scale to inform on the value that otherwise would be shown on the *y*-axes of 1 of the 2 figures.

Figure 4A,B show the tradeoff between the probability of cost-effectiveness (on the *y*-axis in Figure 4A and the color scale in Figure 4B) and expected loss (on the color scale in Figure 4A and the *y*-axis in Figure 4B) that is traded when a strategy is chosen that is not on the frontier. For instance, in Figure 4A, some strategies with a higher probability of cost-effectiveness can be chosen in the WTP regions where the frontier is lowered. From the figure, it can be estimated that the differences in expected loss are quite small in those regions, and depending on the interpretation of the policy maker, this may be an acceptable risk.

Figures 4A and 4B combine the same CEAC and ELC but differ in which method is used for the color scale. One advantage of Figure 4B is that the ELC does not collapse when many strategies are compared. A shortcoming of the heat map is that colors can no longer be used to show which curves correspond with which strategies. This can be solved by labeling the curves in the plots or labeling which strategy is on the frontier in specific WTP regions. These regions border at the incremental cost-effectiveness ratios (ICERs) associated with the strategy number right of the ICER (Appendix 4).

The heat map in Figure 4B shows that many of the curves are blue, which is related to the collapse of the CEAC. The small differences in the expected loss also mean that the risk related to a wrong choice is relatively low and comparable for all strategies, even though the probability of cost-effectiveness may be low. The collapse of the CEAC also affects the discriminatory ability of the heat map in showing which strategies have a higher probability of being cost-effective. This can be resolved with the relaxation of the CEAC (Figure 4C), which results in a broader usage of colors in the plot (Figure 4D). Relaxation is a method that loosens the criterion of what is cost-effective in the CEAC, such that the strategies with almost equal NMB may be counted, and thereby increasing the probabilities. Multiple methods for relaxing the CEAC, namely, ranks, fixed thresholds, and relative differences, are compared in Appendix 3. Relative relaxation performed the best in our comparison and is shown in Figure 4C.

## Discussion

In this study, we identified 9 graphical methods that represent the impact of uncertainty on cost-effectiveness outcomes in analyses comparing multiple strategies. We evaluated these methods using the PSA data sets from 3 case studies. Three of the identified methods (i.e., CEAC,^11,12^ ELC,^17^ and the incremental benefit curve^19^) were assessed as best at communicating uncertainty because they scored highest on interpretability and graphical discriminatory ability. Although both the information about the average loss associated with a decision and the probability of cost-effectiveness of the chosen decision option is relevant for decision makers, none of the identified methods simultaneously provided this information. Consequently, we proposed integrating the information presented in an ELC and CEAC in the form of a single heat map.

Furthermore, we provide 2 open-source tools in R to apply the proposed methods and the identified methods and in Microsoft Excel for the 5 methods that scored highest in our assessment.

Based on our literature search, we found only 1 previous review comparing methods that visualize decision uncertainty. The study by Naveršnik^18^ compared 7 methods by applying them to a PSA data set. Naveršnik compared the sensitivity of methods to output correlations and concluded that methods presenting uncertainty should be sensitive to the underlying output correlations to correctly capture decision uncertainty. We expanded the work of Naveršnik by including 4 new methods in our comparison. The cost-effectiveness plane was not included in this study because it does not capture decision uncertainty, and the ELC is an augmentation of the expected value of perfect information (EVPI) because it shows both the EVPI and the difference in loss relative to the EVPI. In addition, we applied these methods to 3 different PSA data sets to investigate graphical discriminatory ability and interpretability of the methods.

A limitation of our review study is that other relevant methods may have been missed, although we included all methods from guidelines on CEA. Two methods were initially identified in the literature search but were not applicable to a PSA data set and were excluded from the methods comparison. These were Stochastic league tables by Hutubessy et al.^23^ and the Bayesian variant to the CEAC by Moreno et al.^24^ Stochastic league tables address a different question, namely, how to optimize a portfolio with a fixed budget for a range of different strategies with uncertain costs and effectiveness values. Therefore, this requires information on the uncertainty of costs and effectiveness of multiple treatments for different medical conditions. The Bayesian variant on the CEAC can be used to calculate the predictive posterior distribution of the net benefits using a regular PSA data set as its prior. This predictive posterior distribution is subsequently used to make a CEAC. Therefore, the last method does not visualize uncertainty but rather generates an alternative PSA-like data set to be analyzed.

The methods identified in our literature search were applied to 3 PSA data sets. Three features of these methods that are important to adequately convey risk information are interpretability, graphical discriminatory ability, and the usage of a fixed WTP threshold. In our case studies, a threshold of 50,000 €/QALY was used for the methods requiring a fixed WTP. The chosen threshold is not expected to affect graphical discriminatory ability or interpretability. However, the limitation to represent the impact of uncertainty for a fixed WTP is a shortcoming for informing policy makers internationally. Different WTP threshold values are used between countries and even within countries,^25^ and the WTP choice affects which strategy is cost-effective and the risk and losses related to choosing a suboptimal strategy.

Interpretability is a subjective feature of a method and therefore difficult to quantify. Nevertheless, to increase objectiveness, the authors have scored 3 specific questions on features that relate to the understanding of the method and have averaged the scores of the authors and questions.

With regard to graphical discriminatory ability, a serious limitation of many methods is the collapse of curves, meaning that curves overlap with an increasing number of strategies compared. Several articles have discussed the phenomenon of collapsing curves that causes problems with the graphical discriminatory ability of the CEAC. Barton^9^ blamed the heavy penalization of all nonoptimal strategies for the collapse of the CEAC, while this interaction is called “confounding” by Eckermann et al.^26^ Naversňik^18^ argued, on the other hand, that a correlation between strategies causes the collapse. All reviewed methods, with the exception of the return-risk space and isoquants, present overlapping curves when multiple strategies are compared. Methods that use differences in losses between strategies, such as the expected loss curve and the incremental benefit curve, may to a large extent resolve the collapsing of curves. Therefore, these methods are preferred when many strategies are compared.

We have proposed a relaxation of the CEAC, which is a direct solution to the penalization problem as described by Barton and Eckermann.^9,26^ It is worth mentioning that this solution showed less improvement in the PSA data set from Rojnik et al. than in the other data set.^15^ This might be caused by a high correlation level in this data set, as identified by Naveršnik,^18^ and may suggest that a collapse of the CEAC may be caused by a mixture of confounding and correlation when comparing many strategies.

The interpretation of a relaxed CEAC is less straightforward than the traditional CEAC. Strategies that were counted as cost-effective presented expected NMB outcomes close to the optimum and were considered as equally acceptable choice. The cost-effectiveness threshold was set at 99.5% or 99.95% in our examples. Although these threshold values are arbitrary, they represent conservative values because the NMB differences between strategies were smaller than the accuracy in measuring costs and effectiveness. There is currently no solution to what a desirable threshold should be. The threshold should be chosen carefully, and the reasoning behind its choice should be motivated. This may be a topic that future studies could further explore to help reach a consensus on this topic in the future.

In the WTP regions where the CEAF is not the highest curve on the CEAC, there is a discrepancy between the highest probability of cost-effectiveness and the highest expected benefit. In studies with many strategies such as for surveillance scanning or screening, an easily executable schedule with a fixed interval may be preferred if the losses are not too great. Alternatively, a high probability of cost-effectiveness may be preferred, and differences in expected losses of certain strategies can be considered as acceptably small. However, the CEAC alone does not provide a decision maker with the information about expected losses necessary to assess this tradeoff.

For this reason, many articles discussing methods to visualize the impact of uncertainty through a PSA suggest that the uncertainty visualized by the CEAC^11^ should be used in combination with another method to provide information on the differences in the NMB values of strategies. Barton^9^ and Briggs et al.^1^ suggested using the CEAC combined with the EVPI. Eckermann and Willan^26^ suggested using the CEAC and the ELC separately. Fenwick et al.^12^ suggested adding a frontier to the CEAC (i.e., CEAF), and Naveršnik^18^ suggested also using the net benefit density plot in addition to the CEAC to give additional information on the distribution of NMB.

We argue that, in situations where a WTP axis is preferred, the ELC should be used to supplement the CEAC. The CEAF shows the strategies with the maximum expected NMB depending on the WTP, also shown in the ELC. In addition, the connected lowest curves of the ELC are equal to the EVPI curve.^26,27^ Furthermore, the lines of the ELC cross at respective ICERs (see Appendix section 4 for proof). Therefore, the ELC is superior to the EVPI and CEAF.

Our proposed heat map combines the information of the CEAC and ELC in 1 figure, making it easier to see which strategies perform well. In addition, when many strategies are compared, the ELC is less sensitive to collapse than the CEAC is. A shortcoming of the heat map is that colors can no longer be used to show which curves correspond to which strategies, but this can be easily resolved with labels. Therefore, this method may improve the CEAC/CEAF and can be used for decision problems involving both few and multiple strategies.

In situations in which a fixed WTP is acceptable, the return-risk space^20^ and the cumulative rankogram^21^ provide less information than the net benefit density plot^18^ and stochastic dominance^16^ as they use point estimates and ranks instead of distributions. The incremental benefit curve^19^ performs better on graphical discriminatory ability and interpretability than do the net benefit density plot,^18^ the incremental benefit density plot,^19^ and stochastic dominance.^16^ Therefore, the incremental benefit curve should be the preferred method when a fixed WTP is acceptable.

Combinations of the above methods can be used to assess confidence in the results of an analysis and the impact of uncertainty. However, some types of uncertainty, such as structural uncertainty, cannot be investigated using a PSA.^1^ Therefore, to investigate the strengths, weaknesses, and structural choices for a model, using tools such as TRUST would be advisable.^2^ Authors should include these analyses in the discussion of the uncertainty of the model.

Predictions made in cost-effectiveness analyses are surrounded by uncertainty, and risks attached to making a wrong choice should be considered in health policy and medical decision making. This article presents an overview of existing methods for representing uncertainty in multiple-strategy CEAs, with both their strengths and shortcomings. We found that the incremental benefit curve is the most informative method when a fixed WTP is used. Further, we introduced a heat map that integrates the CEAC and the ELC to combine most information and facilitate well-informed decision making.

## Supplemental Material

sj-docx-1-mdm-10.1177_0272989X221100112 – Supplemental material for Methods for Communicating the Impact of Parameter Uncertainty in a Multiple-Strategies Cost-Effectiveness Comparison Click here for additional data file. Supplemental material, sj-docx-1-mdm-10.1177_0272989X221100112 for Methods for Communicating the Impact of Parameter Uncertainty in a Multiple-Strategies Cost-Effectiveness Comparison by Henri B. Wolff, Venetia Qendri, Natalia Kunst, Fernando Alarid-Escudero and Veerle M.H. Coupé in Medical Decision Making

## References

[1] Briggs AH Weinstein MC Fenwick EA , et al. Model parameter estimation and uncertainty analysis: a report of the ISPOR-SMDM Modeling Good Research Practices Task Force Working Group–6. Med Decis Making. 2012;32:722–32. 10.1177/0272989X12458348 22990087

[2] Grimm SE Pouwels X Ramaekers BLT , et al. Development and validation of the TRansparent Uncertainty ASsessmenT (TRUST) tool for assessing uncertainties in health economic decision models. Pharmacoeconomics. 2020;38:205–16. doi:10.1007/s40273-019-00855-9 PMC7081657 31709496

[3] Claxton K Sculpher M McCabe C , et al. Probabilistic sensitivity analysis for NICE technology assessment: not an optional extra. Health Econ. 2005;14:339–47. doi:10.1002/hec.985 15736142

[4] Weinstein MC O’Brien B Hornberger J , et al. Principles of good practice for decision analytic modeling in health-care evaluation: report of the ISPOR Task Force on Good Research Practices–Modeling Studies. Value Health. 2003;6:9–17. doi:10.1046/j.1524-4733.2003.00234.x 12535234

[5] Briggs A Sculpher M Claxton K . Decision Modelling for Health Economic Evaluation. Oxford (UK): Oxford University Press; 2006.

[6] van den Hout WB Kramer GW Noordijk EM , et al. Cost-utility analysis of short- versus long-course palliative radiotherapy in patients with non-small-cell lung cancer. J Natl Cancer Inst. 2006;98:1786–94. doi:10.1093/jnci/djj496 17179480

[7] Fenwick E Steuten L Knies S , et al. Value of information analysis for research decisions—An introduction: report 1 of the ISPOR Value of Information Analysis Emerging Good Practices Task Force. Value Health. 2020;23:139–50. doi:10.1016/j.jval.2020.01.001 32113617

[8] Wolff HB Alberts L Kastelijn EA , et al. Cost-effectiveness of surveillance scanning strategies after curative treatment of non-small-cell lung cancer. Med Decis Making. 2021;41:153–64. doi:10.1177/0272989x20978167 PMC7879224 33319646

[9] Barton P . What happens to value of information measures as the number of decision options increases? Health Econ. 2011;20:853–63. doi:10.1002/hec.1651 20730782

[10] Badampudi D Wohlin C Petersen K . Experiences from using snowballing and database searches in systematic literature studies. In: Proceedings of the 19th International Conference on Evaluation and Assessment in Software Engineering. Association for Computing Machinery; 2015. p 1–10.

[11] van Hout BA Al MJ Gordon GS , et al. Costs, effects and C/E-ratios alongside a clinical trial. Health Econ. 1994;3:309–19. doi:10.1002/hec.4730030505 7827647

[12] Fenwick E Claxton K Sculpher M . Representing uncertainty: the role of cost-effectiveness acceptability curves. Health Econ. 2001;10:779–87. doi:10.1002/hec.635 11747057

[13] Kunst N Wilson ECF Glynn D , et al. Computing the expected value of sample information efficiently: practical guidance and recommendations for four model-based methods. Value Health. 2020;23:734–42. doi:10.1016/j.jval.2020.02.010 PMC8183576 32540231

[14] Rothery C Strong M Koffijberg HE , et al. Value of information analytical methods: report 2 of the ISPOR Value of Information Analysis Emerging Good Practices Task Force. Value Health. 2020;23:277–86. doi:10.1016/j.jval.2020.01.004 PMC7373630 32197720

[15] Rojnik K Naversnik K Mateović-Rojnik T , et al. Probabilistic cost-effectiveness modeling of different breast cancer screening policies in Slovenia. Value Health. 2008;11:139–48. doi:10.1111/j.1524-4733.2007.00223.x 18380626

[16] Stinnett AA Mullahy J . Net health benefits: a new framework for the analysis of uncertainty in cost-effectiveness analysis. Med Decis Making. 1998;18:S68–80. doi:10.1177/0272989X98018002S09 9566468

[17] Eckermann S Briggs A Willan AR . Health technology assessment in the cost-disutility plane. Med Decis Making. 2008;28:172–81. doi:10.1177/0272989X07312474 18356312

[18] Naversnik K . Output correlations in probabilistic models with multiple alternatives. Eur J Health Econ. 2015;16:133–9. doi:10.1007/s10198-013-0558-0 24390145

[19] Bala MV Zarkin GA Mauskopf J . Presenting results of probabilistic sensitivity analysis: the incremental benefit curve. Health Econ. 2008;17:435–40. doi:10.1002/hec.1274 17694580

[20] O’Brien BJ Sculpher MJ . Building uncertainty into cost-effectiveness rankings: portfolio risk-return tradeoffs and implications for decision rules. Med Care. 2000;38(5):460–8. 10.1097/00005650-200005000-00003 10800973

[21] Epstein D . Beyond the cost-effectiveness acceptability curve: the appropriateness of rank probabilities for presenting the results of economic evaluation in multiple technology appraisal. Health Econ. 2019;28:801–7. doi:10.1002/hec.3884 PMC6790661 31050043

[22] Wolff HBVQ Kunst N Alarid-Escudero F Coupe VMH . Methods for communicating the impact of parameter uncertainty in a multiple strategies cost-effectiveness comparison. Available from: https://github.com/HaroldWolff/Methods-Uncertainty/releases/tag/v1.02021 10.1177/0272989X221100112 PMC9452448 35587181

[23] Hutubessy RC Baltussen RM Evans DB , et al. Stochastic league tables: communicating cost-effectiveness results to decision-makers. Health Econ. 2001;10:473–7. 10.1002/hec.614 11466807

[24] Moreno E Girón F Vázquez-Polo F , et al. Complementing information from incremental net benefit: a Bayesian perspective. Health Serv Outcomes Res Methodol. 2010;10:86–99.

[25] Sharma D Aggarwal AK Downey LE , et al. National healthcare economic evaluation guidelines: a cross-country comparison. Pharmacoecon Open. 2021;5(3):349–64. doi:10.1007/s41669-020-00250-7 PMC8333164 33423205

[26] Eckermann S Willan AR . Presenting evidence and summary measures to best inform societal decisions when comparing multiple strategies. Pharmacoeconomics. 2011;29:563–77. doi:10.2165/11587100-000000000-00000 21671686

[27] Alarid-Escudero F Enns EA Kuntz KM , et al. “Time Traveling Is Just Too Dangerous” but some methods are worth revisiting: the advantages of expected loss curves over cost-effectiveness acceptability curves and frontier. Value Health. 2019;22:611–618. doi:10.1016/j.jval.2019.02.008 31104743 PMC6530578

