# Supplementary material for: Methods for Communicating the Impact of Parameter Uncertainty in a Multiple-Strategies Cost-Effectiveness Comparison
Source: Med Decis Making. 2022 May 19;42(7):956–68. doi: 10.1177/0272989X221100112 (PMC9452448; doi:10.1177/0272989X221100112)
Supplement: sj-docx-1-mdm-10.1177_0272989X221100112 – Supplemental material for Methods for Communicating the Impact of Parameter Uncertainty in a Multiple-Strategies Cost-Effectiveness Comparison [file sj-docx-1-mdm-10.1177_0272989X221100112.docx]

**Supplementary materials to**

**Methods for communicating the impact of parameter uncertainty in a multiple strategies cost-effectiveness comparison.**

**Table of Contents**

1. Selected methods for uncertainty representation in multiple strategy cost-effectiveness analysis
   1. *Cost-effectiveness acceptability curve (CEAC)*
   2. *Expected Loss Curve (ELC)*
   3. *Net Benefit Density Plot*
   4. *Incremental Net Benefit Density Plot*
   5. *Stochastic dominance*
   6. *Incremental Benefit Curve*
   7. *Return-risk space*
   8. *Cumulative Rankogram*
   9. *Expected Benefit Plot*
2. Comparison of graphical discriminatory ability of the 9 methods using two additional case studies
3. Relaxation of the CEAC
4. Expected Loss Curves Cross at Incremental Cost-Effectiveness Ratios
5. References

**Appendix 1 – Selected methods for uncertainty representation in multiple strategy cost-effectiveness analysis**

The 9 identified methods that are used to explore the impact of uncertainty employ benefit valiables in their calculations such as net monetary benefit (NMB), net health benefit (NHB), return of investment (ROI), or the difference to the maximum benefit which is defined as a loss.^1-3^ These variables reduce the combination of costs (*C* in €, $, £ or any other currency) and effectiveness (*E* in QALYs, Life Years, or other measures of health impact) to one scale: either costs or effectiveness, to facilitate comparison among alternatives. The willingness to pay (WTP) threshold is used to convert health benefits to monetary benefits or vice versa. Essentially, the WTP thresholds represent the money decision makers are willing to spend for one additional unit of effectiveness.

NMB, NHB, and ROI can be used interchangeably in the mathematical formulae summarizing each of the graphical methods for representing uncertainty in this paper, and are all represented by the Benefit variable (*B*). In the illustrative applications in this paper NMB is used, unless otherwise stated.

NMB is calculated by:

$NMB=B=WTP*E-C$. (1)

*Cost-effectiveness acceptability curve (CEAC)*

The CEAC counts the number of times that strategy $j$ has the maximum benefit $B_{i, max}$ over all PSA simulations $i with i=1,..,N,$ for each WTP value with indexing function $I_{max}$. The probability that $B_{i,j}\left( WTP \right) \mathrm{equals} B_{i,max}(WTP)$ is then estimated by dividing by the total number of PSA simulations $N$. ^4^

$P(B_{i,j}(WTP)=B_{i,max}(WTP))=\frac{1}{N}\sum_{i=1}^{N} I_{max}$, with $I_{max}\left( B_{i,j}\left( WTP \right) \right)=\left\{ \begin{matrix} 0, ifB_{i,j}\left( WTP \right)\neq B_{i,max}\left( WTP \right) \\ 1, ifB_{i,j}\left( WTP \right)=B_{i,max}(WTP). \end{matrix} \right.$ (2)

The CEAC is a line-plot with WTP depicted on the x-axis, and the probability that $B_{i,j}(WTP)=B_{i,max}(WTP)$ on the y-axis. The CEAF determines for each value of WTP which strategy has the maximum expected Benefit value, which is the optimal risk-neutral strategy, and plots this frontier on top of the CEAC. ^5^

*Expected Loss Curve (ELC)*

The ELC^3^ plots loss *L* which is the expected difference between $B_{i,j}$ of the strategy of interest *j* and the maximum Benefit value $B_{i,max}$ over all PSA runs 1 to N, for each value of WTP on the x-axis $E\left[ B_{i,max}-B_{i,j} \right]$. Thus:

$L\left( B_{j}(WTP) \right)=\frac{1}{N}\sum_{i=1}^{N} (B_{i,max}\left( WTP \right)- B_{i,j}\left( WTP \right))$. (3)

Loss is not a probability, but expressed in the same units as the Benefit ($, €, QALYs, LY, etc.).

*Net Benefit Density Plot*

The Net Benefit Density Plot (NBD)^6^ approximates a probability density function (PDF), by plotting a normalized smoothened histogram of the NMB distribution at a fixed WTP value.

To create the smoothened histogram, the NMB values are binned with the width of each bar determined as follows:

$bar width=\frac{B_{max}-B_{min}}{N_{bins}}$. (4)

Here, *B_max_* and *B_min_* are the maximum and minimum NMB of all strategies and simulations, and $N_{bins}$defines the total number of bins used. Subsequently, $B_{min}$and the $bar width$ can be used to calculate the range of each bar. The height of each bar can is then determined as follows:

$bar height=\frac{n_{bar j} * \sigma_{width}}{n_{total} * bar width}$. (5)

Here, $n_{bar j}$ is the number of simulations counted for strategy $j$in the bar, $n_{total}$ is the total number of simulations per strategy, and $\sigma_{width}$is the standard deviation of the strategy expressed in NMB.

Additionally, the plot can be smoothened to reduce the artifacts caused by the histogram approximation method. In our study this is done as follows:

${{bar}_{k} height}_{smooth}=\frac{s * {bar}_{k-1} height + {bar}_{k} height + s * {bar}_{k+1} height}{\left( 2 * s \right) + 1}$. (6)

Here, $s$ is the smoothening parameter, and the bars are indexed with $k=1,2,\ldots,N-1,N$. Alternative methods of smoothening can be used, but these need to ensure the area under the curve sums up to 1.

*Incremental Net Benefit Density Plot*

Incremental benefits (*ΔB*) visualize the differences in NMB between strategies at a fixed WTP value, by calculating for each PSA simulation $i$the additional benefit of the strategy with the highest NMB compared to the second highest benefit, or losses for the other strategies where $B_{i,j}\leq B_{i,max}$. Note that in contrast to the expected loss curve, the losses here are defined as $B_{i,j}-B_{i,max}$ and therefore have a negative value.

${\Delta B}_{i,jx}=\left\{ \begin{matrix} B_{i,j}-B_{i,r=2} & if & B_{i,j}=B_{i,max} \\ B_{i,j}-B_{i,max} & if & B_{i,j}\leq B_{i,max} \end{matrix} \right.$. (7)

Here, *j* is the strategy of interest, and *x* refers to the comparator Benefit value, which is either *B_max_*, or *B_r=2_* which is the second highest NMB in the PSA run.

The Incremental Net Benefit Density Plot (IBD)^6^ approximates a PDF, by plotting a Normalized Smoothened Histogram similar to NMD to visualize the *ΔB* distribution.

*Stochastic dominance*

For Stochastic Dominance (SD), a cumulative probability function (*P*) for each strategy $j$ at a fixed WTP value is approximated by counting PSA simulations that fit the criterion of an indexing function ($I_{\beta}$) divided by the total number of simulations ($N$) as in function 4: ^1, 7^

$P\left( B_{j}\geq\beta| WTP \right)=\frac{1}{N}\sum_{i=1}^{N} I_{\beta}$, with $I_{\beta}\left( B_{j}\left( WTP \right) \right)=\left\{ \begin{matrix} 0, ifB_{j}\left( WTP \right)<\beta\\ 1, ifB_{j}\left( WTP \right)\geq\beta. \end{matrix} \right.$(8)

In de SD plot, $\beta$ is the comparator Benefit value depicted on the x-axis, while the cumulative probability function P is shown on the y-axis. Note that in stochastic dominance plots $\beta$ is shown from high to low values on the x-axis.

*Incremental Benefit Curve*

The Incremental Benefit Curve (IBC)^8^ uses a cumulative probability distribution at a fixed WTP value similar to SD, to plot $\Delta B$ (calculated as in function 7) compared to a difference in NMB ($\Delta\beta$) on the x-axis.

$P\left( {\Delta B}_{jx}(WTP)\geq\Delta\beta|\text{ }WTP \right)=\frac{1}{N}\sum_{i=1}^{N} I_{\Delta\beta}$, with $I_{\Delta\beta}\left( {\Delta B}_{jx}(WTP) \right)=\left\{ \begin{matrix} 0, if{\Delta B}_{jx}(WTP)<\Delta\beta\\ 1, if{\Delta B}_{jx}(WTP)\geq\Delta\beta. \end{matrix} \right.$(9)

The additional benefit values relative to the second highest benefit are per definition all positive, and losses in $\Delta B$ are per definition negative, and these two outcomes are therefore separated on the x-axis, and a horizontal line can be used to separate the two at $\Delta\beta=0$.

*Return-risk space*

The return risk space is a scatterplot of the NMB $B_{j}$ of strategies $j$at a fixed WTP value. The mean and standard deviation of $B_{j}(\mu,\sigma)$ *(*are shown on the x-axis and y-axis respectively.^9^

*Cumulative Rankogram*

The Cumulative Rankogram (CR) is also a cumulative probability function, which compares $B_{j}$ to the *r*-th highest ranked Benefit value ($B_{r}$) for each PSA run $i$ *:*^10^

$P\left( B_{j}(WTP)\leq B_{r}(WTP,r)|\text{ }WTP,r \right)=\frac{1}{N}\sum_{i=1}^{N} I_{cumrank}$, with $I_{cumrank}\left( B_{j}\left( WTP,r \right) \right)=\left\{ \begin{matrix} 0, ifB_{j}(WTP)>B_{r}(WTP,r) \\ 1, ifB_{j}(WTP)\leq B_{r}(WTP,r). \end{matrix} \right.$ (10)

The Cumulative Rankogram plots the cumulative probabilities on the y axis as a function of rank $r$ on the x-axis, resulting in a number of points equal to the number of strategies simulated that are connected with lines.

*Expected Benefit Plot*

The expected benefit plot shows the expected NMB value of each strategy $j$ on the y-axis as a function of WTP on the x-axis. Confidence intervals or prediction intervals can be added to this plot.^1^

**Appendix 2 – Comparison of** **graphical discriminatory ability of the 9 methods using two additional case studies**

PSA datasets of two additional case studies were used to investigate the effect of the number of compared strategies on graphical discriminatory ability. These studies were chosen as they represent the extremes of the spectrum that can still be considered a comparison of many strategies.

The study by Jongeneel et al.^11^ compared the costs and health effects of five different treatment strategies for stage II colon cancer. The strategies included: 1) no adjuvant therapy, 2) the Dutch guidelines (adjuvant treatment in microsatellite stable tumors [MSS] with a pathological T-stage of 4[pT4]) with adherence as observed in clinical practice, 3) adjuvant treatment according to the Dutch guidelines assuming perfect adherence, 4) adjuvant treatment in patients with a MSS tumor in combination with pT4 or a mutation in the (K)RAS or BRAF oncogene, and 5) adjuvant treatment in patients with a MSS status in combination with pT4 and a mutation in the (K)RAS or BRAF oncogene. A five-state Markov cohort model was used in this analysis, with competing risks of death by cancer, death by other causes, and 90-day mortality after surgery. In the study, a PSA was performed in which 82 model parameters were varied.

The study by Wolff et al. ^12^ compared the costs and health effects of 108 surveillance strategies consisting of CT scanning with different intervals after curative treatment of stage I Non-Small Cell Lung Cancer. Multiple competing events were considered, including symptomatic detection, the occurrence of a second primary lung cancer, death due to lung cancer, and death of other causes. A discrete event microsimulation model with underlying tumor growth was used to estimate the time of detectability of tumors on a scan, when tumors became symptomatic, and when the tumor volume reached a lethal size. Twenty-one model parameters were varied using a Latin Hypercube Sampling algorithm to distribute the parameter sets in the PSA efficiently.

*Methods with a WTP axis*

***
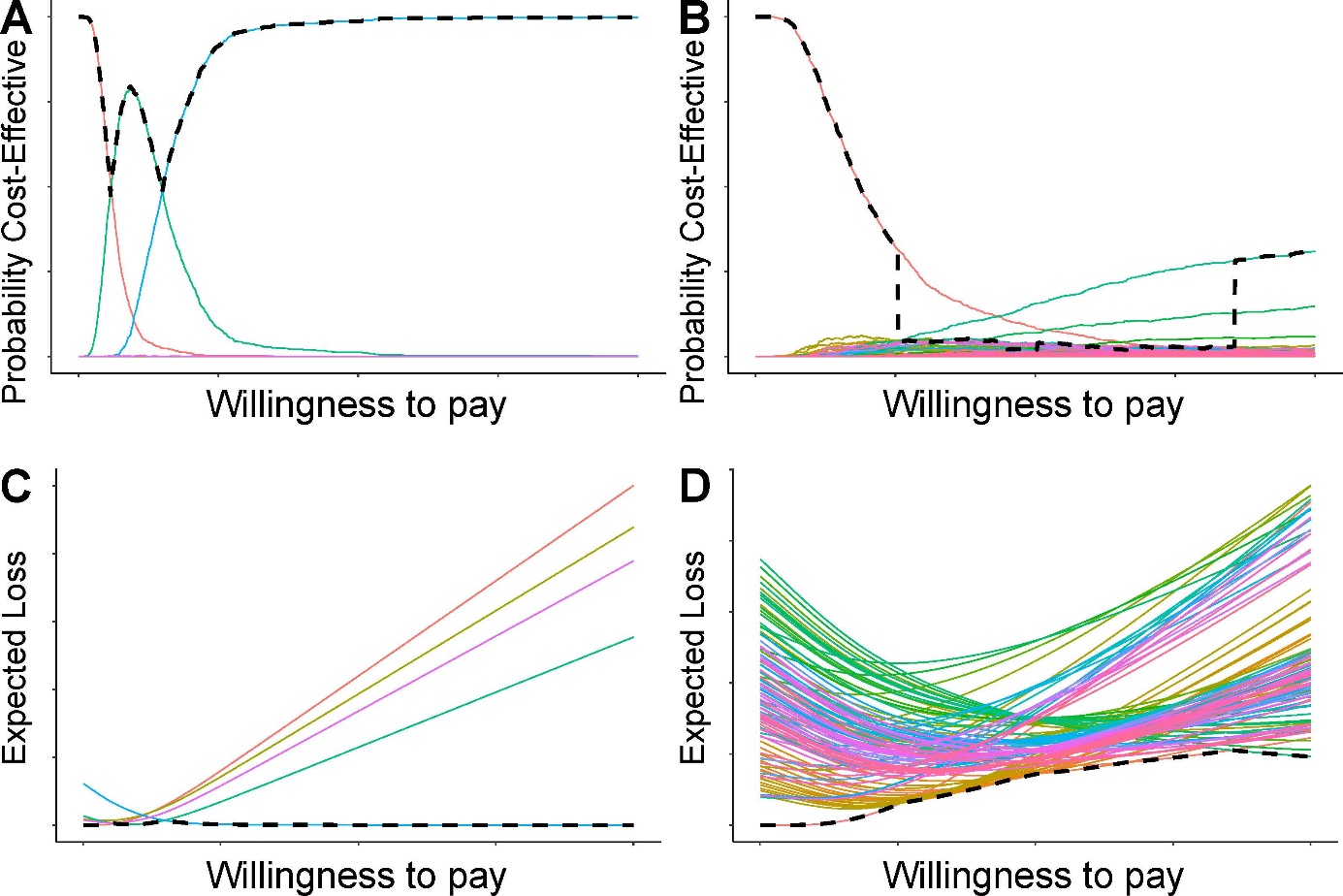
Appendix figure 1*** *Illustrative comparison of methods to communicate the impact of uncertainty with a willingness to pay axis. A and B show the Cost Effectiveness Acceptability Curve (CEAC) and their frontiers (dashed black line)*.^4, 5^ *C and D show the Expected Loss Curves (ELC) and their frontiers (dashed black line).*^3^ *A and C were created using the PSA of Jongeneel et al.^11^ which compares five strategies, while B and D were created using the data of Wolff et al. which compares 105 strategies.^12^ Both methods use the x-axis to depict a range of willingness-to-pay threshold values, whilst the y-axis is used to show probabilities of cost-effectiveness for the CEAC, expected loss values for the ELC. The frontiers show which strategies have the highest expected Net Monetary Benefit.*

Appendix figure 1 shows the effect of the number of compared strategies on graphical discriminatory ability of the Cost Effectiveness Acceptability Curve (CEAC),^4, 5^ and the Expected Loss Curves (ELC).^3^

The graphical discriminatory ability of the CEAC decreases when the number of strategies increase from five to 37 (see also figure 2 in main manuscript) and to 105. This is mainly caused by the collapse of the CEAC, which can be explained by heavy penalization of all non-optimal strategies for each PSA run while these strategies have very similar NMB values.^13^ Unlike the CEAC, the ELC does not collapse and is robust to the number of strategies compared. Of all methods using line-plots to represent uncertainty, the ELC is the least collapsed. For this reason, the ELC was scored with ‘good’ on the graphical discriminatory ability criterion.

The expected benefit plot^1^ is not shown in appendix figure 1 (but it is shown in Figure 2, main manuscript), as it is also completely collapsed for these two cases. Therefore the expected benefit plot scored ‘bad’ on graphical discriminatory ability.

*Methods with a fixed WTP threshold*

***
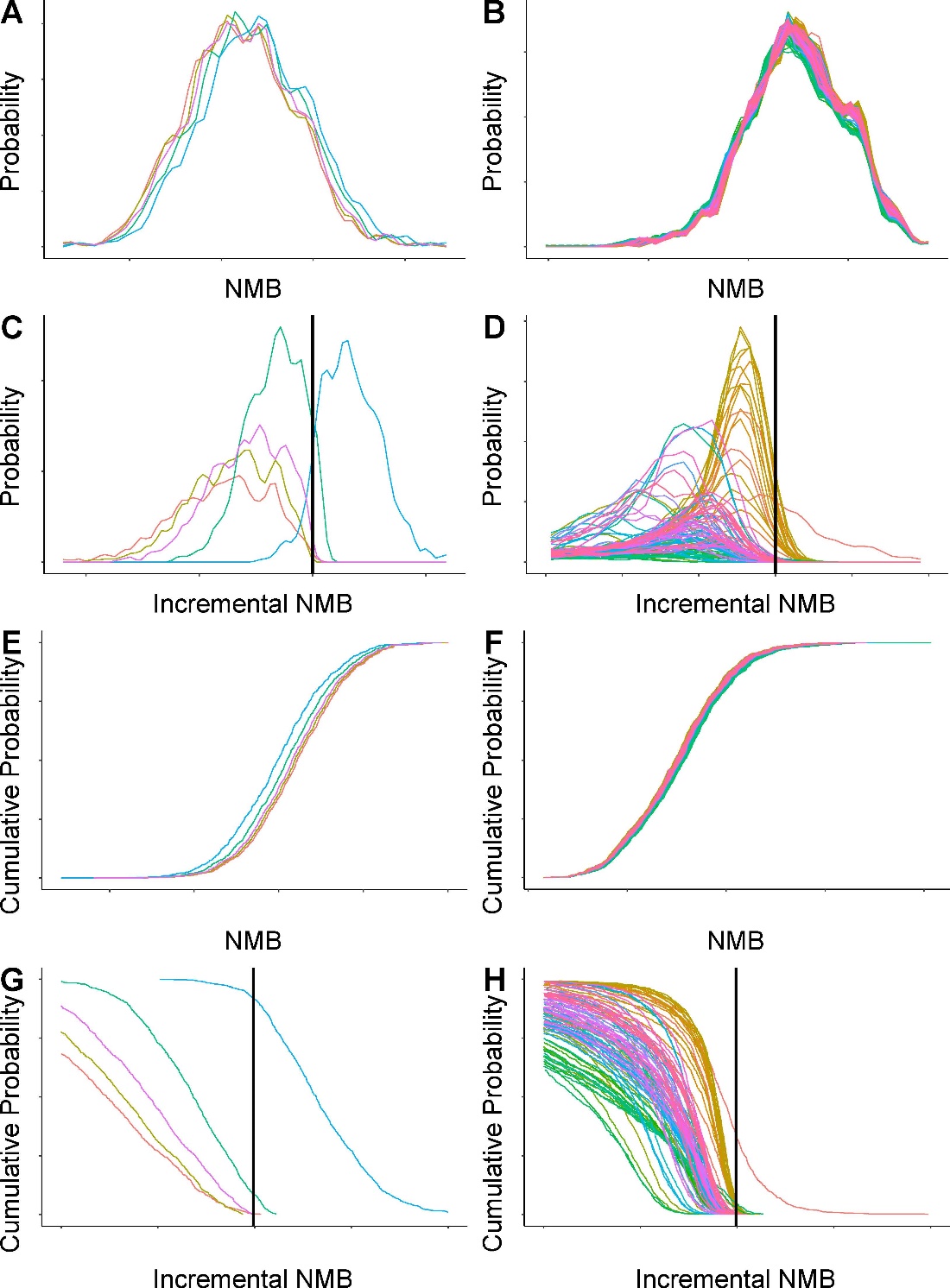
***

***Appendix figure 2*** *Illustrative comparison of methods communicate the impact of uncertainty with a fixed willingness to pay value. The methods are: A and B net benefit density plot^6^, C and D incremental benefit density plot^6^, E and F stochastic dominance plot* ^1^*, G and H incremental benefit curve*^8^. *A, C, E and G were created using the PSA of Jongeneel et al.^11^ which compares five strategies, while B, D, F and H were created using the data of Wolff et al. which compares 108 strategies.^12^*

*To produce these plots the willingness-to-pay threshold was fixed at 50,000 €/QALY for all figures. The probability density plots are normalized smoothened histograms of the NMBs, using 50 bins for A and C, 75 bins for D and 750 bins for F, and the smoothening parameter was set at 0.5 (see appendix 1).*

Appendix figure 2 shows how the number of strategies can affect the graphical discriminatory ability of the net benefit density plot, the incremental benefit density plot, the stochastic dominance plot, and the incremental benefit curve. For all of these methods, graphical discriminatory ability is negatively affected by the number of strategies.

As mentioned in the main paper, the density plots are dependent on smoothening to produce a curve, which makes it impossible to tell if curves that are close actually perform better or not. Incremental benefits on the other hand, do focus on the differences between strategies, and therefore have a positive effect on the graphical discriminatory ability. This is also visible when the numbers of strategies increase. The incremental benefit curve therefore scores the best on graphical discriminatory ability of the methods shown in appendix figure 2.

The return risk space^9^ not included in appendix figure 2 because it uses a scatterplot to visualize uncertainty, which cannot collapse. Therefore it is not sensitive to numbers of strategies, and it scores good on graphical discriminatory ability. The cumulative rankogram^10^ is also not included in appendix figure 2, as the figures of the other two case studies are also uninformative in the same way as in figure 3 of the main paper and scored therefore bad on graphically discriminatory ability.

**Appendix 3 – Relaxation of the CEAC**

A problem with the CEAC^4^ is that the curves collapse when many strategies are compared^6, 13, 14^, because a CEAC depicts the proportion of times that each decision option is the option with the highest NMB over all PSA simulation rounds, similar to “the winner takes it all” principle.^15^ Curves can become graphically indistinguishable when the collapse of the curves is too extreme. This problem can be resolved by a newly proposed option of relaxation of the CEAC, where strategies with comparable NMB to the strategy with the maximum NMB are also counted as cost-effective depending on the threshold used. Here, multiple relaxation options are considered and compared to select the best option.

The CEAC calculates the number of times that strategy $j$ has the maximum NMB $B_{max}$ divided by the total number of PSA simulations $N$. The indexing function $I_{max}\left( B_{j}\left( WTP \right) \right)$, as defined above in equation (2) can be adjusted to relax the criteria of the CEAC. The options compared here are:

Firstly, ranking NMB values within a PSA and counting NMB greater or equal to the NMB value with rank $r$:

$P\left( B_{j}(WTP)\leq B_{r}(WTP) \right)=\frac{1}{N}\sum_{i=1}^{N} I_{rank}$, with $I_{rank}(B_{j}\left( WTP \right))=\left\{ \begin{matrix} 0, ifB_{j}(WTP)>B_{r}(WTP) \\ 1, ifB_{j}(WTP)\leq B_{r}(WTP). \end{matrix} \right.$ (11)

Secondly, relaxation can be achieved using a fixed value of acceptable difference in NMB, $\Delta\beta$:

$P\left( B_{j}(WTP)\geq B_{max}\left( WTP \right)-\Delta\beta\right)=\frac{1}{N}\sum_{i=1}^{N} I_{max}$, with $I_{max}\left( B_{j}\left( WTP \right) \right)=\left\{ \begin{matrix} 0, ifB_{j}\left( WTP \right)<B_{max}\left( WTP \right)-\Delta\beta\\ 1, ifB_{j}(WTP)\geq B_{max}\left( WTP \right)-\Delta\beta. \end{matrix} \right.$ (12)

The third option is relaxation relative to $B_{max}$ with percentage $\theta$.

$P\left( B_{j}\left( WTP \right)\geq B_{max}\left( WTP \right)-\theta*\left| B_{max}\left( WTP \right) \right| \right)=\frac{1}{N}\sum_{i=1}^{N} I_{max}$,

with $I_{max}\left( B_{j}\left( WTP \right) \right)=\left\{ \begin{matrix} 0, ifB_{j}\left( WTP \right)<B_{max}\left( WTP \right)-\theta*\left| B_{max}\left( WTP \right) \right| \\ 1, ifB_{j}\left( WTP \right)\geq B_{max}\left( WTP \right)-\theta*\left| B_{max}\left( WTP \right) \right|. \end{matrix} \right.$ (13)

The reason for this indexing function to count $B_{max}\left( WTP \right)-\theta*\left| B_{max}\left( WTP \right) \right|$ instead of $\theta*B_{max}\left( WTP \right)$, is that all NMB values may be negative for low willingness to pay values. This would result in artefacts at the left side of the relaxed-CEAC.

***
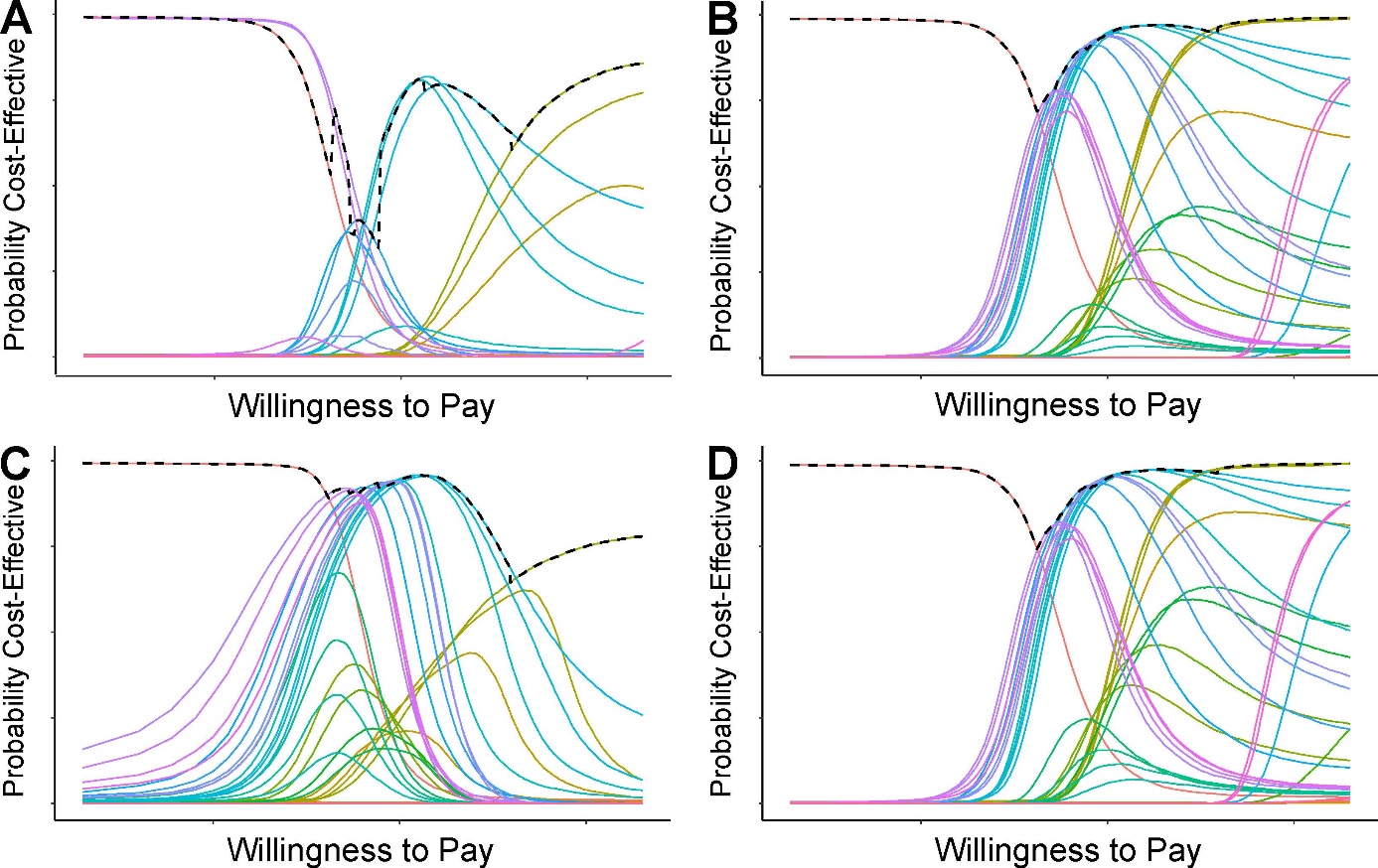
Appendix Figure 3*** *Comparison of methods to relax the CEAC with frontier (dashed lines).*^4, 5^ *Relaxation of the criteria defining strategies as cost-effective will increase the probability of being cost-effective, and reduce the collapse of the curves. In A, strategies with rank ≤ 3 are considered cost-effective. In B strategies with a Net Health Benefit of ≥ the maximum Benefit - 0.01 QALY are considered cost-effective. In C strategies with a Net Monetary Benefit ≥ the maximum Net Monetary Benefit - € 100 are considered cost-effective. In D strategies with a Benefit ≥ 99.5% of the maximum Benefit are considered cost-effective. All figures use the PSA dataset of Rojnik et al.^16^*

The comparison of the different relaxation methods in appendix figure 3 shows that the distance between the CEAF^5^ (black dashed lines) and the highest curves in the relaxed-CEAC are much smaller than in the regular CEAC, with exception of the ranked frontier (A). This observation can be explained by the fact that relaxed criteria are more similar to a risk-neutral decision rule because relaxed criteria are weighted by the size of differences in NMB values. Ranked NMBs are insensitive to the size of the differences in NMBs, which can cause some strategies with very low NMB values to be considered cost-effective.

Appendix figure 3B and C shows that fixed differences in NMBs and Net Health Benefits (NHB) have different effects on the shape of the curves. The reduction of the collapse is stronger for NMB at low values of WTP , while NHB has more reduction of the collapse at higher values of WTP. The reason for this difference is explained by the calculation method of the NMB and NHB. $NMB=WTP*E-C$ and $NHB=E-C/ WTP$. Here $E$ represents effectiveness and $C$ costs. The consequence is that NHB is driven by effectiveness for high values of WTP, and NMB is driven by costs for low values of WTP. This means that the value of WTP has different effects on the difference in NMB or NHB, which makes the usage of a fixed Benefit difference criterion difficult to interpret.

In contrast to fixed differences, relative differences in NMBs in Appendix figure 3D have a relaxing effect to the curves that is not sensitive to the value of WTP. The criterion of a relative difference to the maximum Benefit is either met or not, irrespective of the usage of NMB or NHB, which makes this relaxation approach more straightforward in terms of interpretation than using a fixed difference.

It should also be noted that different threshold values may be used in Appendix figure 3 depending on the number of strategies compared and the correlation within the PSA dataset. For this reason, it is subjective what a good threshold may be. The relaxation methods were also tested on the other case studies of Jongeneel et al. and Wolff et al. (data not shown). In all these cases, conservative criteria were sufficient to have a large effect on the shape of the CEAC.

The presented relaxed CEAC options account for the fact that in certain decision problems with many potential strategies, such as in the context of screening, many strategies may differ only marginally and the choice needs to be partly based on considerations of practicality and public support. Overall, the relaxed CEAC increased the graphical discriminatory ability compared to the normal CEAC, at the cost of a decreased interpretability because the interpretation of a relaxed criterion is less straightforward. Relaxed CEACs should therefore be used when the CEAC is collapsed, and this is considered as a separate figure to distinguish a group of cost-effective strategies.

**Appendix 4 – Expected Loss Curves Cross and Incremental Cost-Effectiveness Ratios**

Here we show the mathematical proof that for any two strategies for which the expected loss curves (ELC) cross, the WTP value at the crossing corresponds to the incremental cost-effectiveness ratios (ICER) of the strategy that is optimal to the right of that crossing relative to the strategy to the left.

If the ELC curves of two strategies cross, it means that there is one specific WTP value at which their Expected Loss is equal. According to function 3 this means that:

${Loss}_{1}={Loss}_{2}=\frac{1}{N}\sum_{i=1}^{N} B_{i,max}\left( WTP \right)- B_{i,1}\left( WTP \right)=\frac{1}{N}\sum_{i=1}^{N} B_{i,max}\left( WTP \right)- B_{i,2}\left( WTP \right)$. (13)

Both share the same maximum values benefit values, which means that:

$\frac{1}{N}\sum_{i=1}^{N} B_{i,1}\left( WTP \right)=\frac{1}{N}\sum_{i=1}^{N} B_{i,2}\left( WTP \right)$ (14)

should also be true. *B* is the Net Monetary Benefit, and replacing $B$ with function 1 in function 14 results in:

$\frac{1}{N}\sum_{i=1}^{N} WTP*E_{i,1}-C_{i,1}=\frac{1}{N}\sum_{i=1}^{N} WTP*E_{i,2}-C_{i,2}$. (15)

Summations can be simplified to:

$WTP*\frac{1}{N}\sum_{i=1}^{N} E_{i,1}-\frac{1}{N}\sum_{i=1}^{N} C_{i,1}=WTP*\frac{1}{N}\sum_{i=1}^{N} E_{i,2}-\frac{1}{N}\sum_{i=1}^{N} C_{i,2}$, and everything can be reordered to:

$WTP=\frac{\frac{1}{N}\sum_{i=1}^{N} C_{i,2}-\frac{1}{N}\sum_{i=1}^{N} C_{i,1}}{\frac{1}{N}\sum_{i=1}^{N} E_{i,1}-\frac{1}{N}\sum_{i=1}^{N} {CE}_{i,2}}=\frac{1}{N}\sum_{i=1}^{N} \frac{C_{i,2}-C_{i,1}}{{E_{i,1}-E}_{i,2}}$. (16)

This is the expected ICER of strategy 1 and 2.

**5 - References**

1. Stinnett AA and Mullahy J. Net health benefits: a new framework for the analysis of uncertainty in cost-effectiveness analysis. *Med Decis Making* 1998; 18: S68-80. 1998/05/05. DOI: 10.1177/0272989X98018002S09.

2. Sendi P, Al MJ and Zimmermann H. A risk-adjusted approach to comparing the return on investment in health care programs. *Int J Health Care Finance Econ* 2004; 4: 199-210. 2004/07/28. DOI: 10.1023/B:IHFE.0000036046.80562.06.

3. Eckermann S, Briggs A and Willan AR. Health technology assessment in the cost-disutility plane. *Med Decis Making* 2008; 28: 172-181. 2008/03/22. DOI: 10.1177/0272989X07312474.

4. van Hout BA, Al MJ, Gordon GS, et al. Costs, effects and C/E-ratios alongside a clinical trial. *Health Econ* 1994; 3: 309-319. 1994/09/01. DOI: 10.1002/hec.4730030505.

5. Fenwick E, Claxton K and Sculpher M. Representing uncertainty: the role of cost-effectiveness acceptability curves. *Health Econ* 2001; 10: 779-787. 2001/12/18. DOI: 10.1002/hec.635.

6. Naversnik K. Output correlations in probabilistic models with multiple alternatives. *Eur J Health Econ* 2015; 16: 133-139. 2014/01/07. DOI: 10.1007/s10198-013-0558-0.

7. Leshno M and Levy H. Stochastic dominance and medical decision making. *Health Care Manag Sci* 2004; 7: 207-215. 2005/01/15. DOI: 10.1023/b:hcms.0000039383.54736.65.

8. Bala MV, Zarkin GA and Mauskopf J. Presenting results of probabilistic sensitivity analysis: the incremental benefit curve. *Health Econ* 2008; 17: 435-440. 2007/08/19. DOI: 10.1002/hec.1274.

9. O'Brien BJ and Sculpher MJ. Building uncertainty into cost-effectiveness rankings: portfolio risk-return tradeoffs and implications for decision rules. *Med Care* 2000: 460-468.

10. Epstein D. Beyond the cost-effectiveness acceptability curve: The appropriateness of rank probabilities for presenting the results of economic evaluation in multiple technology appraisal. *Health Econ* 2019; 28: 801-807. 2019/05/03. DOI: 10.1002/hec.3884.

11. Jongeneel G, Greuter MJE, van Erning FN, et al. Model-based effectiveness and cost-effectiveness of risk-based selection strategies for adjuvant chemotherapy in Dutch stage II colon cancer patients. *Therap Adv Gastroenterol* 2021; 14: 1756284821995715. 2021/04/01. DOI: 10.1177/1756284821995715.

12. Wolff HB, Alberts L, Kastelijn EA, et al. Cost-Effectiveness of Surveillance Scanning Strategies after Curative Treatment of Non-Small-Cell Lung Cancer. *Med Decis Making* 2021; 41: 153-164. 2020/12/16. DOI: 10.1177/0272989x20978167.

13. Barton P. What happens to value of information measures as the number of decision options increases? *Health Econ* 2011; 20: 853-863. 2010/08/24. DOI: 10.1002/hec.1651.

14. Eckermann S and Willan AR. Presenting evidence and summary measures to best inform societal decisions when comparing multiple strategies. *Pharmacoeconomics* 2011; 29: 563-577. 2011/06/16. DOI: 10.2165/11587100-000000000-00000.

15. Barton GR, Briggs AH and Fenwick EA. Optimal cost-effectiveness decisions: the role of the cost-effectiveness acceptability curve (CEAC), the cost-effectiveness acceptability frontier (CEAF), and the expected value of perfection information (EVPI). *Value Health* 2008; 11: 886-897. 2008/05/21. DOI: 10.1111/j.1524-4733.2008.00358.x.

16. Rojnik K, Naversnik K, Mateović-Rojnik T, et al. Probabilistic cost-effectiveness modeling of different breast cancer screening policies in Slovenia. *Value Health* 2008; 11: 139-148. 2008/04/03. DOI: 10.1111/j.1524-4733.2007.00223.x.
